# Diagnosis and Treatment of Alcoholic Liver Disease and Its Complications

**Published:** 2003

**Authors:** Luis S. Marsano, Christian Mendez, Daniell Hill, Shirish Barve, Craig J. Mcclain

**Affiliations:** Luis S. Marsano, M.D., is a professor of internal medicine; Christian Mendez, M.D., is a gastroenterology fellow; Daniell Hill, M.D., is an associate professor of internal medicine; Shirish Barve, Ph.D., is an associate professor of internal medicine; and Craig J. McClain, M.D., is vice chair for research in the Department of Internal Medicine and professor of internal medicine and pharmacology and toxicology; all authors are associated with the University of Louisville Medical Center, Louisville, Kentucky

**Keywords:** alcoholic liver disorder, diagnosis, disease complication, treatment method, lifestyle, nutritional deficiency, vitamin therapy, drug therapy, propylthiouracil, colchicines, corticosterone, alternative medical treatment, S-adenosylmethionine, ascites, peritonitis, kidney disorder, esophageal varix, pentoxifylline, hepatorenal syndrome

## Abstract

Alcoholic liver disease (ALD) is a serious and potentially fatal consequence of alcohol use. The diagnosis of ALD is based on drinking history, physical signs and symptoms, and laboratory tests. Treatment strategies for ALD include lifestyle changes to reduce alcohol consumption, cigarette smoking, and obesity; nutrition therapy; and pharmacological therapy. The diagnosis and management of the complications of ALD are important for alleviating the symptoms of the disease, improving quality of life, and decreasing mortality.

The liver is one of the largest and most complex organs in the body. It performs multiple functions, including the production of proteins and enzymes, detoxification, metabolic functions, and the regulation of cholesterol and blood clotting. Because the liver is primarily responsible for alcohol metabolism, it is especially vulnerable to alcohol-related injury.

Alcoholic liver disease (ALD) is a serious and potentially fatal consequence of drinking alcohol. ALD encompasses three conditions: fatty liver, alcoholic hepatitis, and cirrhosis (see figure 1). Fatty liver (i.e., steatosis), the most common alcohol-induced liver disorder, is marked by the excessive accumulation of fat inside the liver cells. Alcoholic hepatitis is inflammation and more severe injury of the liver, in which the body’s immune system responds to and causes liver damage. In cirrhosis, normal liver cells are replaced by scar tissue (i.e., fibrosis), and consequently the liver is unable to perform many of its usual functions.

Cirrhosis and alcoholic hepatitis often coexist and cause substantial morbidity and mortality. For example, studies from the Department of Veterans Affairs (VA) demonstrate that patients with both cirrhosis and alcoholic hepatitis have a death rate of greater than 60 percent over a 4-year period, with most of the deaths occurring in the first year (Chedid et al. 1991). Thus, the mortality rate for ALD is greater than that of many common types of cancer such as colon, breast, and prostate. This article examines the issues of diagnosing and treating ALD and the complications of this disease.

## Diagnosis of Alcoholic Liver Disease (ALD)

The diagnosis of ALD is established by a history of habitual alcohol intake of sufficient duration and quantity, together with physical signs and laboratory evidence of liver disease. Alcohol dependence is not a prerequisite for the development of ALD, and ALD can be difficult to diagnose because patients frequently minimize or deny alcohol abuse. In addition, there may be no evidence of ALD from the physical exam, and laboratory abnormalities may not specifically point to ALD.

Ambulatory patients with alcoholic fatty liver often are asymptomatic. Patients with alcoholic hepatitis may be asymptomatic, have only enlarged liver (i.e., hepatomegaly), or have full-blown alcoholic hepatitis with tender hepatomegaly, jaundice, fever, accumulation of fluid in the abdominal cavity (i.e., ascites), nervous system effects such as confusion and personality change (i.e., hepatic encephalopathy), anorexia, and fatigue. Other signs may include high white blood cell counts resembling those seen in leukemia (i.e., leukemoid reactions) and the rapid deterioration of kidney function (i.e., hepatorenal syndrome). Even in the absence of cirrhosis, the main vein that brings blood from the intestine and stomach into the liver (i.e., the portal vein) may come under increased pressure because of scarring of the liver, resulting in portal vein hypertension.

Ten to 20 percent of patients with alcoholic hepatitis develop cirrhosis, and up to 70 percent of alcoholic hepatitis patients go on to develop cirrhosis each year (Bird and Williams 1988). Women are at higher risk for developing cirrhosis, as are people who continue drinking or have severe alcoholic hepatitis (Pares et al. 1986). Some patients with alcoholic hepatitis who abstain still may develop cirrhosis, but others will have complete clinical and histologic recovery.

Patients with early stage alcoholic cirrhosis with no complications (i.e., well-compensated) may be asymptomatic and have normal physical exams and normal routine blood tests of liver function and injury. In other patients, alcoholic fatty liver or alcoholic hepatitis often coexist and may be accompanied by hepatomegaly, an enlarged spleen (i.e., splenomegaly), or both. In cirrhotics with severe alcoholic hepatitis, hepatomegaly or splenomegaly may be the dominant feature; in other patients, the signs and symptoms of portal vein hypertension (e.g., ascites and engorged veins [varices] in the esophagus) may predominate. As the disease advances, the liver decreases in size, the left hepatic lobe becomes more prominent, and the entire liver has a hard and nodular consistency. Splenomegaly of varying degrees is frequent.

In later stage cirrhosis with complications (i.e., decompensated disease), patients may have muscle wasting, ascites, and the adaptation of smaller vessels to handle increased blood flow (i.e., venous collateral circulation). Other common signs are small star-shaped vessels (i.e., spider angiomata) on the skin of the upper torso, blotchy redness on the palms (i.e., palmar erythema), and contracture of the palm tissue, causing the ring and pinky finger to bend into the palm (i.e., Dupuytren’s palmar contracture). Enlargement of the parotid gland (one of the salivary glands) and the lacrimal (tear) glands often is seen. Enlargement of the fingertips may be found in patients who develop a problem with the way blood passes through the lungs, resulting in blood not being properly oxygenated. Other physical signs, which may be found during examination with a flexible fiberoptic instrument (i.e., endoscopy), include changes in the stomach lining that occur with portal hypertension, as well as engorged veins in the esophagus, stomach, or another part of the gastrointestinal tract, which expand as a consequence of increased pressure in the blood flow of the venous system. Patients with hepatic encephalopathy may have slow reaction times and muscle tremors causing involuntary jerking of the hands.

ALD cannot be diagnosed based on any of the physical signs and symptoms alone. Laboratory tests often assist in the diagnosis of ALD. Almost all patients will have elevated liver enzymes. The level of the enzyme aspartate aminotransferase (AST) will exceed that of alanine aminotransferase (ALT), but both will be below 300 international units per milliliter (IU/ml). When the ratio of AST to ALT is greater than 2, the most likely diagnosis is ALD. In some studies, more than 80 percent of patients attain this ratio.

Elevated blood levels of the liver enzyme gamma glutamyltransferase (GGT) indicate heavy alcohol use and liver injury. This test has greater ability to correctly test positive (i.e., sensitivity) but less ability to correctly test negative (i.e., specificity) than AST or ALT tests. Of the three enzymes, GGT is the best indicator of excessive alcohol consumption, but because GGT is present in many organs and because some drugs raise GGT levels, high GGT levels are not necessarily an indicator of alcohol abuse.

Chronic alcohol consumption also may be associated with abnormally high triglyceride levels (i.e., hypertriglyceridemia), high blood levels of uric acid (i.e., hyperuricemia), and low amounts of potassium (i.e., hypokalemia) and magnesium, as well as an elevated index of red blood cell size (i.e., mean corpuscular erythrocyte volume [MCV]). Hyperuricemia and hypertriglyceridemia often normalize with abstinence, and hypokalemia normalizes with adequate potassium replacement. Elevated MCV often is found in people who ingest more than 50 grams of alcohol per day,^1^ with sensitivity of 27 to 52 percent and specificity of 85 to 90 percent. The blood protein known as carbohydrate-deficient transferrin frequently is used to detect current or recent alcohol abuse, especially consumption in excess of 60 grams per day (Nilssen et al. 1992; Litten et al. 1995), but there are no ideal tests to identify continuing alcohol intake.

An increased number of white blood cells (i.e., leukocytosis) and decreased number of platelets (i.e., thrombocytopenia) are common in alcoholic hepatitis. Thrombocytopenia may be transitory, but in patients with concomitant cirrhosis, it is persistent. Markers of severe alcoholic hepatitis or cirrhosis include elevated levels of bilirubin (a yellow-orange substance generated in the liver), prolonged time required for a blood sample to clot (i.e., prothrombin time [PT]), and a low level of the main circulating protein in the bloodstream (i.e., albumin), which is synthesized by the liver (i.e., hypoalbuminemia). The most commonly used prognostic index in alcoholic hepatitis is Maddrey’s Discriminant Function (DF), which is calculated by this equation:

4.6 [PT(patient)-PT(control)]+total bilirubin (mg/dl).

If this value exceeds 32, the mortality rate during a current hospitalization may exceed 50 percent (Maddrey et al. 1978; Carithers et al. 1989). There also is evidence that blood concentrations of proteins (i.e., cytokines) that promote inflammation—such as tumor necrosis factor alpha (TNF–α), interleukin–6, and interleukin–8—correlate with mortality in patients with alcoholic hepatitis (McClain et al. 1993), but levels of these cytokines are not determined in routine clinical practice.

Liver biopsy mainly is used to clarify atypical cases, to better define the contribution of alcohol in patients with possible non-alcohol-related coexisting conditions (e.g., hepatitis C, use of lipid-lowering medications), and to determine the severity of liver disease. Many laboratories are conducting research to evaluate biomarkers or identifier proteins for detecting ongoing alcohol abuse and ALD. The importance of genetic variations in alcoholism and ALD among individuals also is under active investigation. New tests may provide novel ways of identifying alcohol abuse, susceptibility to liver injury, and mechanisms of liver injury, and of detecting and monitoring liver injury.

## Treatment of ALD

Treatment strategies for ALD include lifestyle changes to reduce alcohol consumption, cigarette smoking, and obesity; nutrition therapy; pharmacological therapy; and possibly liver transplantation (see textbox). (Liver transplantation is discussed in detail in the article by Anantharaju and Van Thiel in this issue.)

**Therapy For ALD**

- Lifestyle modification (decreased alcohol use, smoking, obesity)
- Appropriate nutrition/nutritional support
- Use of pentoxifylline or prednisone for alcoholic hepatitis
- Advice on complementary and alternative medicine (e.g., silymarin or SAMe) for cirrhosis
- Transplantation in selected abstinent patients with severe (i.e., end-stage) disease.

### Lifestyle Changes

Abstinence from alcohol is vital in order to prevent further liver injury, scarring, and possibly liver carcinoma; it appears to benefit patients at every stage of the disease. Fatty liver is reversible with abstinence. Although evaluations of the effects of abstinence on the progression of ALD are few and have involved retrospective, nonrandomized trials, virtually all these studies have shown beneficial effects of abstinence (Powell and Klatskin 1968; Merkel et al. 1996). Patients with either compensated or decompensated cirrhosis benefit from abstinence. Thus, all patients with ALD should be encouraged to abstain from alcohol consumption. Newer medications to facilitate abstinence, such as naltrexone and acamprosate, have been shown to be effective in some chronic alcoholics, but no large multicenter studies have evaluated these medications in patients with ALD.

Many people who drink alcohol also smoke cigarettes. In European studies, fibrosis worsens more rapidly in ALD patients who smoke cigarettes (Klatsky and Armstrong 1992; Corrao et al. 1994). Patients with hepatitis C who drink also deteriorate faster if they smoke cigarettes (Pessione et al. 2001). Cigarette smoking causes oxidative stress, a condition that arises when an overabundance of free radicals is present in the body, which may be a factor leading to accelerated liver disease in smokers.

Obesity is associated with the development of fatty liver and nonalcoholic steatohepatitis, a disorder that is histologically identical to alcoholic hepatitis. Body mass index has been shown to be an independent risk factor for the development of ALD (Raynard et al. 2002). An increasingly large subset of ALD patients are obese, with alcohol intake as a source of excess and empty calories (that is, having no nutritional value). Thus, as with many other gastrointestinal disorders (e.g., gastro-esophageal reflux disease), the initial approach to treating ALD is lifestyle modification to reduce alcohol consumption, cigarette smoking, and obesity.

### Nutrition Therapy

Malnutrition is prevalent in alcoholic hepatitis and cirrhosis, especially in end-stage ALD, and can range from deficiency in individual nutrients (e.g., zinc, folate) to global protein–calorie malnutrition.

Researchers at the VA Cooperative Studies Program have conducted some of the most extensive studies of nutritional status in patients with alcoholic hepatitis (Mendenhall et al. 1995). The first of these studies (VA Cooperative Study 119) demonstrated that virtually every patient with alcoholic hepatitis had some degree of malnutrition. Patients had an average alcohol consumption of 228 grams per day, with almost 50 percent of energy intake coming from alcohol. The severity of liver disease generally correlated with the severity of malnutrition.

**Figure 2:**
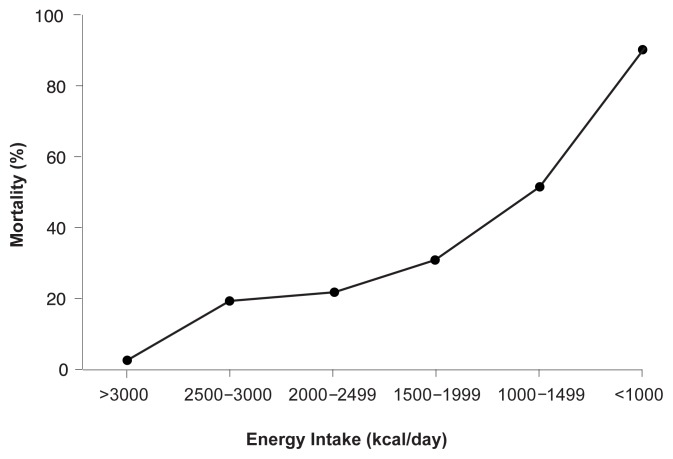
Inadequate nutrition was directly related to mortality in the Veterans Health Administration studies in patients who had moderate to severe alcoholic hepatitis. It is not known whether providing nutrients directly into the gastrointestinal tract (that is, enteral feeding) to those patients with inadequate caloric intake would have improved their survival. NOTE: Kcal/day = Kilocalories per day.

A followup VA study on alcoholic hepatitis (VA Cooperative Study 275) found similar results. In both of these studies, patients were given a balanced 2,500-kilocalorie hospital diet, monitored carefully by a dietitian, and were encouraged to follow it. In the second study, patients in the treatment group also received a liquid nutritional supplement high in three amino acids that help to stimulate protein synthesis (which was administered as an oral food supplement), as well as the anabolic steroid oxandrolone. In neither study were patients fed by tube if voluntary oral intake was inadequate (probably a design flaw, in retrospect). Voluntary oral food intake correlated in a stepwise fashion with 6-month mortality data—that is, almost all patients who voluntarily consumed more than 3,000 kcal per day still were living at the end of the 6-month period, whereas more than 80 percent of those consuming less than 1,000 kcal per day died within that time (see figure 2) (Mendenhall et al. 1995). Moreover, the degree of malnutrition correlated with the development of serious complications such as encephalopathy, ascites, and hepatorenal syndrome (Mendenhall et al. 1995).

Interest in nutrition therapy for cirrhosis was stimulated when Patek and colleagues (1948) demonstrated that a nutritious diet improved the 5-year outcome of patients with alcoholic cirrhosis compared with patients consuming an inadequate diet. Several recent studies have found improved outcomes in cirrhosis patients who were given nutritional support. Hirsch and colleagues (1993) demonstrated that outpatients receiving a nutritional support product (1,000 kcal, 34 grams protein) through a feeding tube (i.e., enteral nutritional support) had significantly improved protein intake and significantly fewer hospitalizations. These investigators subsequently gave enteral nutritional support to outpatients with alcoholic cirrhosis and observed an improvement in nutritional status and immune function (Hirsch et al. 1999).

VA Cooperative Study 275 found that the combination of an anabolic steroid and an oral nutritional supplement reduced the mortality rate of patients who had moderate protein–energy malnutrition (Mendenhall et al. 1995). Those with severe malnutrition did not significantly benefit from the therapy, possibly because their malnutrition was so advanced that no intervention, including nutrition, could help.

Kearns and colleagues (1992) showed that patients with ALD who were hospitalized for treatment and given an enteral nutritional supplement via tube feeding had significantly improved serum bilirubin levels and liver function. Moreover, a major randomized study of enteral nutrition versus steroids in patients with alcoholic hepatitis showed similar overall initial outcomes, as well as fewer long-term infections in the nutrition group (Cabre et al. 2000). This important study suggests that aggressive nutritional support is as effective as treatment with prednisone (an immunosuppressive medication), with its potential complications (e.g., infections, diabetes, osteoporosis), in hospitalized patients with alcoholic hepatitis.

Thus, traditional nutritional supplementation clearly improves nutritional status and, in some instances, hepatic function and other outcome indicators in alcoholic hepatitis and cirrhosis.

### Pharmacological Therapy

Although ALD remains a major cause of morbidity and mortality in the United States, there is no FDA-approved therapy for either alcoholic cirrhosis or alcoholic hepatitis. However, several drugs have been used “off label.”

#### Propylthiouracil (PTU)

Orrego and colleagues (1987) examined long-term PTU therapy in more than 300 patients with various types of liver disease, including ALD. In this study, mortality was reduced by nearly 50 percent in patients receiving PTU. A recent review (Rambaldi and Gluud 2002) evaluated PTU therapy for ALD, including alcoholic fatty liver, alcoholic fibrosis, alcoholic hepatitis, and cirrhosis. Combining the results of six randomized clinical trials (710 patients), these researchers did not find any significant effects of PTU versus placebo on mortality from all causes or liver-related mortality, complications of liver disease, or liver histology. The negative result of this review limits enthusiasm for PTU as a treatment for ALD.

#### Colchicine

Because of its antifibrotic effects, colchicine has been suggested as a treatment for ALD. Initial positive studies by Kershenobich and colleagues (1988) led to a large VA Cooperative Study evaluating colchicine therapy in patients with alcoholic cirrhosis. Results showed no beneficial effect on either overall mortality or liver-related mortality (Morgan et al. 2002). A recent smaller study from Europe also showed no beneficial effects of colchicine (Cortez-Pinto et al. 2002). Thus, despite initial enthusiasm and biochemical rationale for use of this drug, it does not appear to be effective in ALD treatment.

#### Pentoxifylline (PTX)

Akriviadis and colleagues (2000) evaluated PTX in a prospective, randomized, double-blind clinical trial in patients with severe alcoholic hepatitis. Forty-nine patients received PTX and 52 received placebo (vitamin B_12_) for 4 weeks. PTX treatment improved survival: 12 PTX patients died (24.5 percent), compared with 24 placebo patients (46 percent). PTX also decreased the percentage of deaths caused by hepatorenal syndrome. Renal failure was the cause of death of 6 of the 12 PTX-treated patients who died (50 percent), compared with 22 of the 24 control patients who died (92 percent). Multivariate analysis revealed that age, kidney test results for the waste product creatinine at randomization, and treatment with PTX were independent factors associated with survival.

#### Corticosteroids

Although corticosteroids are the most extensively studied form of therapy for alcoholic hepatitis, their role remains limited. The rationale for steroid use is to decrease the immune response and the proinflammatory cytokine response. Most randomized studies have supported the use of corticosteroids in moderate to severe alcoholic hepatitis (Carithers et al. 1989), but a large multi-center VA study yielded negative results (Mendenhall et al. 1984). Steroids have been found to be effective against severe acute alcoholic hepatitis by most meta-analyses, including the most recent study by Mathurin and colleagues (2002). These investigators reported significantly improved survival at 28 days (85 percent vs. 65 percent) in severely ill alcoholic hepatitis patients having a DF greater than 32. This survival advantage may extend to 1 year but not 2. Independent prognostic factors associated with survival at 28 days in this meta-analysis were steroid treatment, age, and serum creatinine levels. Patients with infections, gastrointestinal bleeding, and many other common complications were excluded from these studies.

Most investigators agree that if corticosteroids are to be used, they should be reserved for patients with severe liver disease (i.e., DF greater than 32), and possibly those with hepatic encephalopathy. Steroids have well-documented side effects, including enhancing risk of infection, which already is substantial in patients with alcoholic hepatitis. Thus, a major disadvantage to corticosteroids is that they cannot be used by many patients with alcoholic hepatitis.

#### Complementary and Alternative Medicine (CAM) Agents

CAM agents have had some success in patients with liver disease and are widely used. Research demonstrates a strong rationale for using many of these CAM agents, such as S-adenosylmethionine, but shows that others may have no efficacy or may even cause harm, including liver injury.

## Diagnosis and Treatment of the Complications of ALD

Proper diagnosis and management of the complications of ALD are vital to decreasing the deterioration of illness, improving quality of life, and possibly decreasing mortality. The complications of ALD reviewed here are: ascites (accumulations of fluid in the abdominal cavity), infections in this fluid that develop without any apparent cause (i.e., spontaneous bacterial peritonitis [SPB]), hepatorenal syndrome, and esophageal varices.

### Ascites

Ascites is one of the most common complications of advanced liver disease and generally indicates a poor prognosis and a high likelihood of death. Approximately 8 of every 10 patients who have ascites in the United States have it as a consequence of cirrhosis of the liver. It is estimated that 3 of every 10 patients who have cirrhosis without complications will develop ascites within the next 5 years. In patients with ascites, the likelihood of death in the following year is approximately 50 percent, compared with 10 percent for cirrhosis patients without complications. Ascites cases can be classified as easily treatable or refractory and difficult to control. Patients with the latter type are likely to develop hepatorenal syndrome (Garcia-Tsao 2001).

**Figure 3:**
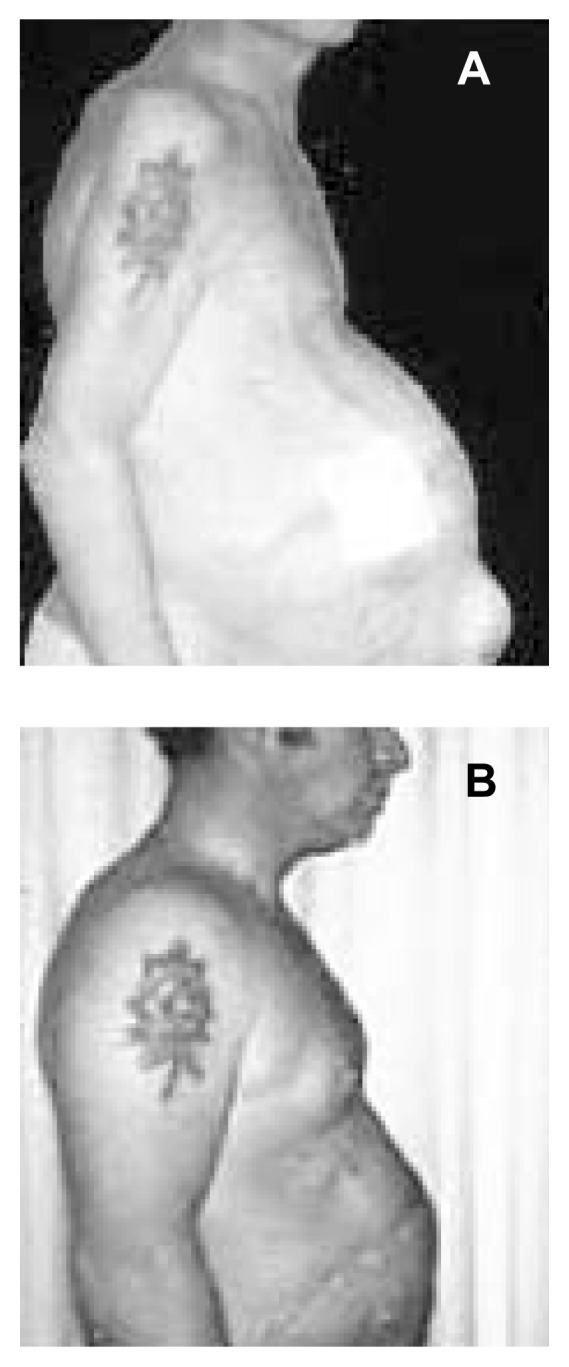
**(A)** Patient with alcoholic cirrhosis who shows ascites, an umbilical hernia, and wasting of muscle. **(B)** After 2 years of abstinence and appropriate nutrition, the patient gained back muscle mass and his ascites improved.

Ascites leads not only to aesthetic changes in body shape but, more importantly, to:

- Increased risk of spontaneous infection of the ascitic fluid.
- Development of abdominal hernias where the abdominal wall muscle becomes weakened and part of the abdomen protrudes (sometimes to the extreme of spontaneous rupture) (see figure 3).
- Difficulty breathing because of pressure of the abdomen on the respiratory muscles.
- Decreased food intake, with progressive malnutrition.
- Decreased physical activity with consequent loss of muscle mass.

Treating ascites does not seem to prolong life in cirrhotic patients but improves the quality of life and protects patients from spontaneous infections of the fluid, which are associated with high death rates.

Patients who develop ascites for the first time, those with ascites who are admitted to the hospital because of illness, those who have difficult-to-control ascites, and those who develop symptoms because of tense ascites (i.e., the abdomen is tight with ascites)—all should have the fluid removed for diagnostic evaluation and to lessen discomfort in the abdomen. Because these patients are at high risk of infection in the fluid and because the fluid accumulation may be a consequence not only of liver disease but also of other associated disorders, this fluid should be thoroughly evaluated. Evaluation should include a cell count (Runyon 1997, 1998) as well as determinations of total protein and albumin. At around the same time, a blood sample should be checked to measure the amount of albumin in order to facilitate interpretation of the findings on the ascitic fluid. For patients who have ascites because of portal vein hypertension, determining serum albumin concentration–ascitic albumin concentration (SAAG) is recommended. SAAG is calculated by subtracting the albumin concentration of the ascitic fluid from the albumin concentration of a serum specimen obtained on the same day. Patients with SAAGs of 1.1 grams per deciliter (g/dl) or higher may have cirrhosis or alcoholic hepatitis, among other conditions.

To control the formation of ascites, patients must eliminate more sodium than they acquire through diet. Patients with cirrhosis and ascites tend to retain sodium very efficiently, and most patients need to have dietary sodium restricted to less than 2 grams per day (88 mEq [milliequivalent, or the number of grams of solute dissolved in one milliliter of solution]). To prevent additional ascites, a patient who is following a 2-gram sodium diet needs to lose at least 78 mEq sodium per day through the urine in addition to the 10 mEq that are lost regularly through the skin. Research has shown that if a person is eliminating a ratio of sodium to potassium through urine that is greater than 1, that person will be eliminating at least 78 mEq sodium per day in urine. To be able to eliminate this amount of sodium, most patients need to take diuretics.

Patients who have no swelling of the extremities should not lose more than half a kilogram of weight per day in order to control ascites. Those who have swelling in the extremities may be able to lose up to 1 kilogram per day. Fluid restriction is not required except in patients with very low sodium concentration in the blood. So that patients will not become dehydrated, the dose of diuretics is adjusted based on the patients’ response to the medication (i.e., it is titrated) to obtain a zero balance of sodium once the ascites has been controlled (Runyon 1997, 1998; Andy and Ke-Qin 2001). Once a patient has developed ascites, he or she is at high risk of death. For that reason, liver transplantation should be contemplated if the patient is a suitable candidate.

### Spontaneous Bacterial Peritonitis (SBP)

Of patients hospitalized with ascites, between 10 percent and 30 percent will develop SBP. This infection is thought to occur either by spontaneous passage of normal bacteria that reside in the gut into the ascitic fluid, or by seeding of bacteria into the blood from a distant source (e.g., a urinary infection or lung infection), leading to growth of this bacteria in the ascitic fluid (O’Grady et al. 2000).

Patients who have low protein concentrations in the ascites (less than 1.5 g/dl) are at higher risk of developing SBP. Once a patient has had an episode of SBP, there is a 7 in 10 chance that a new episode will occur within the next year. With every episode, 2 to 3 of every 10 patients will die from complications of the infection, and only 3 of every 10 are expected to survive for 2 years (Garcia-Tsao 2001).

The diagnosis of SBP is made when high numbers of a type of white blood cell that is especially protective against bacterial infections (i.e., polymorphonu-clear cells [PMN]) are found in the ascitic fluid (i.e., in excess of 250 per ml). Patients are diagnosed as having SBP if bacteria are found in the ascitic fluid, but the concentration of bacteria in the fluid is extremely low—an estimated 1 bacterium per milliliter of fluid—so that the bacteria cannot be seen by examining the fluid under the microscope. In order to have a reasonable chance of getting a positive culture, the fluid should be injected at the bedside into blood culture bottles specially designed to recover small amounts of bacteria. This technique will detect the vast majority of infections. If improper techniques are used (e.g., sending the fluid to the laboratory to be placed on a culture plate), chances of proper diagnosis decrease to 4 out of 10.

Patients with SBP may not have symptoms, or manifestations of the infection may appear to be unrelated to the abdominal cavity. For example, SBP patients may have confusion, changes in kidney function, poorly controlled ascites, or overall progressively deteriorating health. Despite the fact that more than half of the patients with SBP complain of some degree of abdominal pain or discomfort, the physical exam of the abdomen usually is completely benign.

Usually only one type of bacteria appears in the culture of patients who have SBP. Clinicians should suspect the possibility of a secondary peritonitis (e.g., some intra-abdominal perforation or abscess formation in the abdomen) if (1) multiple kinds of bacteria are recovered from the culture, or (2) the patient develops an infection consistent with SBP but in the presence of total protein concentration in the ascitic fluid of more than 1.5 g/dl, or (3) the patient fails to respond promptly to proper antibiotic therapy. A secondary infection also should be suspected if direct examination of fluid under the microscope shows bacteria because, as mentioned, the concentration of bacteria in SBP is so low that bacteria should not be detectable by microscopic examination (Andy and Ke-Qin 2001; Garcia-Tsao 1992).

Patients who develop SBP are at extremely high risk of developing kidney dysfunction and hepatorenal syndrome. Expansion of the volume within the blood vessels (i.e., the intravascular volume) using intravenous albumin infusions has been shown to decrease the frequency of hepatorenal syndrome and, for that reason, improves survival rates in patients who develop SBP (Sort et al. 1999). Because of concerns about kidney toxicity, it is important to avoid antibiotics or other medications that may exacerbate kidney damage. Current therapy for SBP includes use of the antibacterial drug cefotaxime.

Patients at special risk for SBP are those who are hospitalized and have a total protein concentration in the ascitic fluid that is less than 1.5 g/dl, those who have gastrointestinal bleeding from any source, and those who have had previous episodes of SBP (Andy and Ke-Qin 2001). Prophylactic therapy (i.e., antibiotic treatment) is indicated for all these groups of patients.

### Hepatorenal Syndrome

Hepatorenal syndrome is the deterioration of kidney function in patients who have acute or chronic liver failure but otherwise healthy kidneys. This disorder may occur spontaneously but more often is the result of an infection, an episode of gastrointestinal bleeding, or overly aggressive use of diuretics. There are two types of hepatorenal syndrome: Type I conveys the highest risk of death and develops rapidly over a couple of weeks; Type II progresses more slowly, usually over several months. The diagnosis of hepatorenal syndrome requires fulfillment of all of the following criteria:

- Deterioration of kidney function demonstrated by a concentration of creatinine in the blood of more than 1.5 mg/dl or a decrease in creatinine clearance to less than 40 milliliters per minute.
- No evidence of dehydration, no exposure to a drug or infection that causes kidney toxicity, and absence of severe low blood pressure (shock).
- Failure to respond to discontinuation of diuretics or to treatment with 1.5 liters of saline solution to expand blood volume.
- No evidence of obstruction of urine flow or primary kidney disease, as demonstrated by ultrasound exam.
- Little or no protein in the urine.

The majority of patients who will develop hepatorenal syndrome will first develop diuretic-resistant ascites, usually with very low sodium in the urine (i.e., less than 10 mEq/L). In general, among patients who have ascites, the risk of developing hepatorenal syndrome is 2 in 10 during the first year and 4 in 10 during the first 5 years. Among patients with Type I hepatorenal syndrome, the estimated mortality without appropriate therapy is 80 percent after 2 weeks and 90 percent after 10 weeks (Gines et al. 1993).

Hepatorenal syndrome is thought to result from a severe contraction of the artery that feeds the kidney, which may occur as a response to excessive relaxation of the vessels in the rest of the body and a relatively low volume of blood inside the vascular system. Thus, the first approach to treatment of this disorder is to try to expand vascular volume and then to increase the degree of contraction of vessels other than the kidney artery.

Several strategies have been used to reverse hepatorenal syndrome. The first involves intravenous infusions of albumin (to expand vascular volume), followed by administration of ornipressin, a medication that increases the contraction of most of the vessels in the body (Guevara et al. 1998*a*). Similar effects with fewer complications have been obtained using intravenous albumin with terlipressin, which causes vessel contraction more safely (Gines et al. 2001; Moreau et al. 2002).

In the United States, neither ornipressin nor terlipressin is available, and the most popular intervention for hepatorenal syndrome is to expand blood volume using intravenous albumin, then to administer midodrine and octreotide to regulate vascular contraction (Angeli et al. 1999). For patients who cannot tolerate medication by mouth, intravenous albumin can be used, followed by continuous infusion of norepinephrine (Duvoux et al. 2002). With the latter two regimens, beneficial effects usually can be seen by day 10 of therapy and, if not successful, the treatment usually is discontinued after 15 days. Patients who do respond can complete at least 2 weeks of therapy, and after that, therapy can be discontinued, usually without deterioration of kidney function.

Other therapies are being used, including the transjugular intrahepatic portal systemic shunt (TIPS), in which a long catheter is inserted via the jugular vein in the neck, advanced into a hepatic vein and then into a large branch of the portal vein in the liver. Using an inflatable balloon-tipped catheter tube, the section between the portal vein branch and the hepatic vein is widened and then kept open (stented) with a cylindrical wire-mesh stent (Guevara et al. 1998*b*). This helps to lower the increased pressure in the portal vein. In addition, hepatorenal syndrome has been treated using high doses of antioxidants (Holt et al. 1999). More recently, a system known as MARS (molecular absorbent recirculating system) has been used (Mitzner et al. 2000); with this treatment, a patient’s blood is transported to a filter, where it is mixed with albumin, which carries the toxins out of the blood.

Although these treatments are available, hepatorenal syndrome will very likely recur, and patients should be moved quickly in the direction of possible liver transplantation.

### Esophageal Varices

Cirrhosis causes an increase in pressure in the fine net of blood vessels inside the liver. This pressure is transmitted back to the portal vein and onto the veins that form it. Because of the high pressure on the veins forming the portal vein and the portal vein itself, blood tries to escape, forming new collateral veins (i.e., the increased pressure causes very small varices to grow larger). Many of these veins are localized superficially inside the esophagus and the upper part of the stomach. When these veins engorge because of increased pressure, they are called varices.

Varices in the esophagus and stomach are present in about half of all cirrhosis patients. Each year, 5 percent to 20 percent of patients with cirrhosis experience the formation of varices. Once varices have been formed, 5 percent to 15 percent become large varices (Garcia and Sanyal 2001). Large varices are highly likely to rupture. Patients with more severe liver disease tend to have more varices, and their varices tend to be larger. Spontaneous rupture of these varices and severe bleeding occur when the pressure on the portal vein, expressed as the difference between the pressure in the portal vein and the pressure in the vein that drains out of the liver (i.e., the portal pressure gradient), is greater than 12 mm of mercury. Large varices have a 2-year risk of bleeding spontaneously in approximately 3 of every 10 cases. Small varices have a risk of only 1 in 10 (Garcia and Sanyal 2001). Because it is difficult to determine that varices are developing, endoscopic evaluation of the esophagus and stomach is recommended at 1- to 2-year intervals in patients with cirrhosis to determine whether varices have formed and if they are large enough to require treatment to prevent bleeding.

Varices in the esophagus that are large or show evidence of high risk for bleeding should be considered for treatment to prevent bleeding (i.e., primary prophylaxis), such as the use of a class of drugs that block the action of the involuntary nervous system on the heart, and therefore relieve stress on the heart (i.e., nonselective beta blockers). An analysis of 11 prospective studies evaluating the effectiveness of nonselective beta blockers at preventing initial variceal bleeding showed that beta blockers decreased the risk of first bleed by at least 40 percent (25 percent in control subjects vs. 15 percent in the treatment group) after a median of 2 years (Garcia-Tsao 2001). Isosorbide mononitrate and isosorbide dinitrate, which dilate both the veins and the arteries, have been evaluated for this purpose but have proven to be ineffective as single agents. Adding low doses of these medications, however, to a regimen of nonselective beta blockers may be of some help.

**Figure 4:**
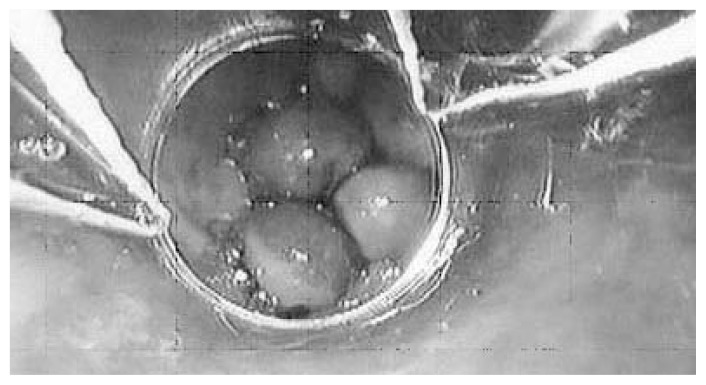
View of varices in the esophagus, a consequence of liver disease, using endoscopy. These dilated veins are being banded to prevent gastrointestinal bleeding.

Treatment in the form of ligation of varices, in which elastic bands are placed around varices using a device attached to the end of the endoscope, also has proven to be effective but usually is reserved for patients who have large varices and cannot tolerate beta blockers (see figure 4). As mentioned, patients with esophageal varices may bleed spontaneously. Approximately half of these patients will stop bleeding spontaneously. The frequency of death for each of these episodes is 3 in 10. It is estimated that bleeding will recur within 6 weeks in 4 of 10 patients who have esophageal variceal bleeding. Thus, treatment of bleeding from esophageal varices has two components: immediate treatment to control the present bleed and additional treatment to prevent consequent bleeding (i.e., secondary prophylaxis).

In the United States the initial medical intervention for suspected variceal bleeding is to administer intravenous octreotide, which has been shown to safely and effectively control variceal bleeding by regulating vascular contraction and decreasing the pressure in the portal venous system (Garcia and Sanyal 2001; D’Amico et al. 1999). The method that has proven to be the best in controlling acute variceal hemorrhage is endoscopic banding of the esophageal varices. When banding is not possible, injecting the varices with scarring substances also can be used. Because cirrhotic patients with gastrointestinal bleeding are at high risk of developing serious infections, a prophylactic 7-day course of the antibiotic norfloxacin is recommended. A patient who has had the first episode of variceal bleeding faces an 8-in-10 chance of bleeding again in the next 3 years. In addition, treatment with nonselective beta blockers, in a manner similar to primary prophylaxis, should be given to patients who are not already receiving these drugs. Patients who cannot tolerate the medication, or for whom beta blockers are contra-indicated, can be treated with repeated banding of the varices at 10- to 14-day intervals.

Patients who rebleed despite therapy with beta blockers and endoscopy should be considered for the TIPS procedure or surgery performing a distal spleno-renal shunt, thereby decreasing the high pressure of the veins by connecting the high-pressure vessels to the inferior vena cava system, which is a low-pressure system (i.e., it carries oxygen-poor blood to the heart from the lower half of the body). Despite the fact that TIPS is more effective than endoscopic therapy in decreasing rebleeding from esophageal varices (19 percent rebleeding with TIPS vs. 47 percent with endoscopic therapy), 1 in 3 of these patients will develop hepatic encephalopathy after TIPS, and this intervention does not affect survival rates. Because of the problems with encephalopathy and the cost of TIPS, this approach usually is reserved as rescue therapy. Liver transplantation is, of course, definitive therapy and should be considered for all of these patients.

## Conclusions

In summary, it now is generally possible to accurately diagnose ALD, and new biomarkers or identifier proteins for detecting ongoing alcohol abuse and ALD are being investigated, as is the role of genetics in ALD. Although there are no FDA-approved therapies for alcoholic liver disease, lifestyle changes, nutritional support, and “off-label” therapies such as PTX can improve outcome. Similarly, new therapies for complications are improving quality of life and, in some cases, even reducing mortality rates.

## Figures and Tables

**Figure 1:**
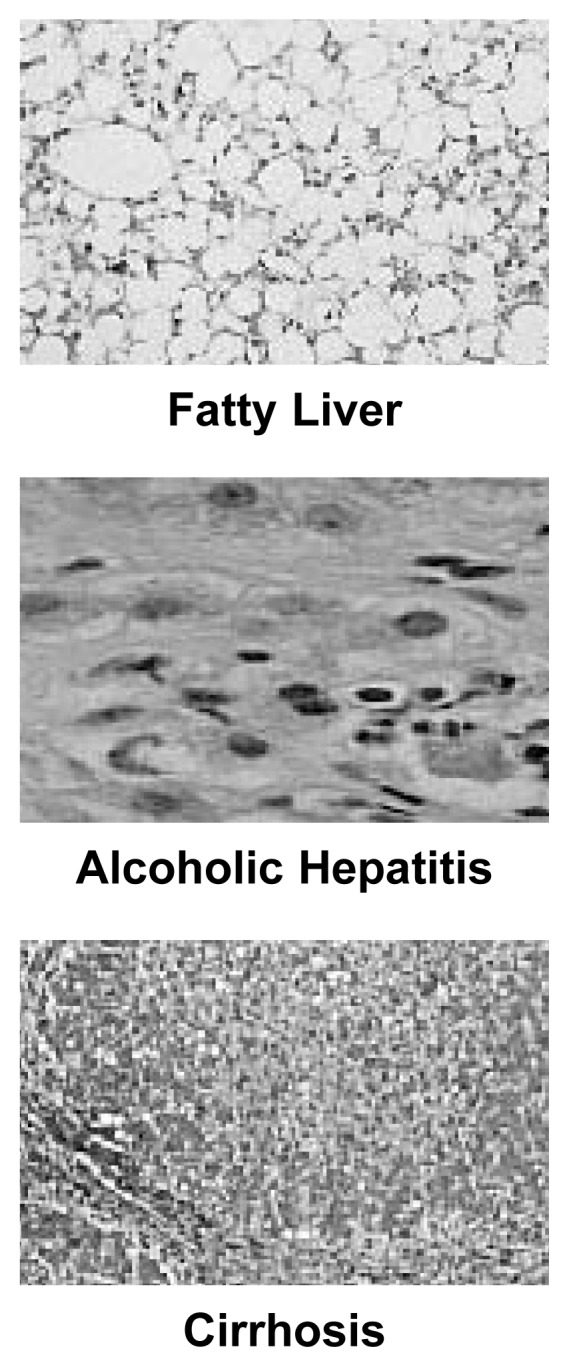
Biopsies of alcoholic liver disease showing how a patient can progress from fatty liver and alcoholic hepatitis to cirrhosis.

## Footnotes

1 In the United States, a drink is considered to be 0.5 ounces (oz) or 15 grams of alcohol, which is equivalent to 12 oz (355 milliliters [ml]) of beer, 5 oz (148 ml) of wine, or 1.5 oz (44 ml) of 80-proof distilled spirits (USDHHS 2000).
